# The rate of species extinction in declining or fragmented ecological communities

**DOI:** 10.1371/journal.pone.0285945

**Published:** 2023-07-12

**Authors:** John M. Halley, Stuart L. Pimm

**Affiliations:** 1 Department of Biological Applications and Technology, School of Health Sciences, University of Ioannina, Ioannina, Greece; 2 Nicholas School of the Environment and Earth Sciences, Duke University, Durham, North Carolina, United States of America; The Clifton Institute, UNITED STATES

## Abstract

Loss of habitat can take many forms, ranging from the fragmentation of once-continuous habitat to the slow erosion of populations across continents. Usually, the harm leading to biodiversity loss is not immediately obvious: there is an extinction debt. Most modelling research of extinction debt has focussed on relatively rapid losses of habitat with species loss happening in response afterwards. In this paper, using a niche-orientated community model we compare and contrast two different mechanisms and find contrasting patterns of extinction debt. From small fragments, we typically see the rapid initial loss of many species, followed by a slower loss of species on larger timescales. When we consider slow incremental declines of population sizes, we find initially a slow rate of extinction which subsequently increases exponentially. In such cases, the delayed extinctions may go undetected initially both because the extinctions may be small relative to background randomness and because rate itself is not constant and takes time to reach its maximum.

## Introduction

Human actions harm species in many ways. Some act quickly–we destroy a species’ habitat, for example–and the consequences immediate and obvious. Other consequences are pernicious–delayed, so that the full impact of our actions may not be immediately obvious. Species may become extinct only after decades. In short, there will be an extinction debt [1, 2]. Our purpose is to understand the timing of these delayed consequences, so that we can better anticipate and manage them. An extinction debt can emerge in two important ways but until now little attention have been given to this distinction. One class of problems is when land-use change reduces once-continuous habitats to small, isolated fragments. Their smaller populations are prone to eventual extinction [3–5]. Another class includes the slow erosion in the numbers of individuals in a community, perhaps through pollution, over-harvesting, or global warming. Again, the full impacts may be postponed.

### The loss of species from habitat fragments

Roughly two-thirds of the world’s terrestrial species live in tropical moist forests [6, 7]. Large fractions of threatened species live in a subset of these forests [8], conveniently referred to as biodiversity hotspots. The salient features of these landscapes are how little habitat remains and how much of that is in fragments. Numerous studies show that small fragments hold fewer species than large ones and so presumably have lost more and may lose more in the future [4]. We know less about how quickly fragments lose species, since this requires counts over time.

An exception involves the experimentally established fragments north of Manaus, Brazil [3, 9, 10]. Even here, the study is relatively short-term. Not surprisingly, the smaller fragments lose more species and lose them more quickly. How many more species should become extinct in the long-term? Answering this question is deceptively important. First, how might we extrapolate from a decade following habitat isolation to how fast fragments will lose species in the future? Ferraz et al. and Brooks et al. showed half the species went extinct from forest fragments from 1 to 100 km^2^ on a time scale of decades [9, 11]. Diamond examined areas of ~10,000 to 100,000 km^2^ isolated by Pleistocene sea-level rises and estimated they had lost half their species on a scale of 10,000 years [12]. Certainly, it might be just a matter of the different sizes of the areas. It could also be a matter of the temporal patterns of species loss. If, after very long intervals, species are still going extinct, it does not necessarily mean the initial loss of species was slow. Conversely, how might what we observe in the short-term help us understand the eventual long-term losses from fragments? Possibly, there might have been a rapid, transient loss of many species–of the kind Brooks et al. document–then a very much slower loss of other species, of the kind Diamond documents.

### Species suffering a gradual slow decline

Many studies describe falling populations either regionally or more locally for a diverse range of taxa: birds in the UK [13], bees [14], moths [15], farmland invertebrates [16], reptiles [17], amphibians [18], and predatory fish [19]. This may arise from a simple mechanism such as overharvesting or a complex suite of factors. For example, the decline of the UK’s population of wild birds is attributed primarily to land-use change (which includes agriculture, development, road building and others) but clearly is exacerbated by other things such as poaching, accidents, pollution etc. Either way, we need to anticipate whether there will be extinctions and, if so, how fast will they occur.

An extension is when species move pole-wards or up-slope, as the climate warms. Species may remain a long time in their now presumably too-hot environments before finally succumbing. Some case studies show species moving more slowly than expected [20] or expanding north without retreating from their southern reaches [21]. Perhaps, some lowland or southern populations are lingering in eventually doomed habitats. Quite generally, delayed extinctions may give the false impression that the loss of species following habitat destruction is not so severe [5]. The same is true in situations with climate change. When species are extending their ranges northwards but lagging in their retreat from the south, the overall, but false, impression is a situation that is improving. In both cases, our interest is in the speed at which the consequent but delayed extinctions happen. Understanding delayed extinctions give us a chance to anticipate them better and perhaps prevent them. If restoration of corridors to reconnect isolated fragments is possible, then how quickly must we act [22]?

### Existing models, their limitations, and how to address them

The question of how fast we expect to lose species is a deceptively difficult one. Consider the loss from fragments. The simplest expectation that there should be an exponential decay in species numbers must surely be wrong. It assumes that all species have the same risk of extinction, so that the number lost in an interval is proportional to the number of species remaining. In fact, species differ greatly in their risks. Most obviously, they differ greatly in their abundances. Rarity is the most important predictor of extinction risk across spatial scales from small islands [23] to globally [24, 25]. We must understand how extinction risk varies among species that may differ substantially in their abundance. In addition, species numbers vary over time, sometimes bringing once abundant populations to low numbers where they are at heightened risk of extinction. We must also understand the nature of population variability.

Various models have been deployed to study delayed extinction. Most of these have been empirical, fitting plausible functions to data without reference to community mechanisms that specify those functions [1, 26]. An explicit description of the process of extinction using a community model should yield greater insights. Many of these approaches are based on the generalised Lotka-Volterra system [27–29]. While improved computing power has circumvented many of the obvious technical difficulties, one is still left with the task of assigning interaction strengths for the model ecological community. Another approach is taken in two studies of extinction debt [30, 31] that used Hubbell’s neutral model [32] of biodiversity. In the neutral model, all species numbers perform a random walk under demographic stochasticity–the vagaries of individual species’ births and deaths. These create relatively small or slow changes in numbers for large populations, while imposing a powerful risk of extinction when populations are small. While the neutral model has yielded insights, there are practical and theoretical criticisms of it [33, 34]. The neutral model has no environmental stochasticity–the external pressures that can cause substantial mortality for even an abundant species and which lead to large year-to-year variation in numbers. The neutral model has no niches either, a significant limitation since an occasional bad year for one species may create opportunities for another. Finally, while it does generate a species-area relationship (SAR), the SAR generated by a neutral model is distinctly different from the Arrhenius SAR, that is found at most scales of interest [35]. Many other models describe biodiversity of the ecological community [36–39] but the number of dynamic community models is much smaller.

A community model that reflects the uncertainties of environmental stochasticity and includes niche constraints would be an important addition to ecological theory. In this study, we use a recently-published model [40] to address this need. The model [40] builds on the concept of the multi-dimensional hypercube to describe the ecological niche [41]. It continues to be a standard approach in ecology [42, 43]. The approach combines a very simple version of the hypercube with dynamics, to arrive at a model with the essential attributes commonly observed in ecological populations and communities. In contrast to the neutral model of Hubbell [32], environmental pressures on that niche drive the dynamics of the populations.

This study aims primarily to examine how the problem of extinction debt plays out differently in two scenarios of community population loss. The first a sudden loss of community size, resulting from a sudden loss of habitat, for example. The second a slow leaching of the community population over time, which might result from a long-term change or deterioration of the environment. In both cases, the loss of biodiversity happens because of an environmental stress that is mediated through the loss of individuals from the community. We use our recently-published dynamic community model [40] to explore theoretically the expected pattern of extinctions in time for the two scenarios and to make predictions. To parameterise the model, and to anchor it in reality, we use data from two corresponding cases of community depletion. The representative scenario for the first case is from near Manaus in Brazil, where rapid deforestation removed most of the habitat, leaving behind a series of fragments. The bird community of these fragments was studied by Pimm and others from 1979 to 1992 [3, 9, 10]. By contrast, wild-bird community of Great Britain has been well studied for many years and provides a scenario of a gradually declining community. A third common source of data for our parameterization is the Global Population Dynamics Database (GPDD) [59] which we use to quantify the natural variance growth in time of animal populations.

## Methods

### The model’s essential features

**Fig 1:**
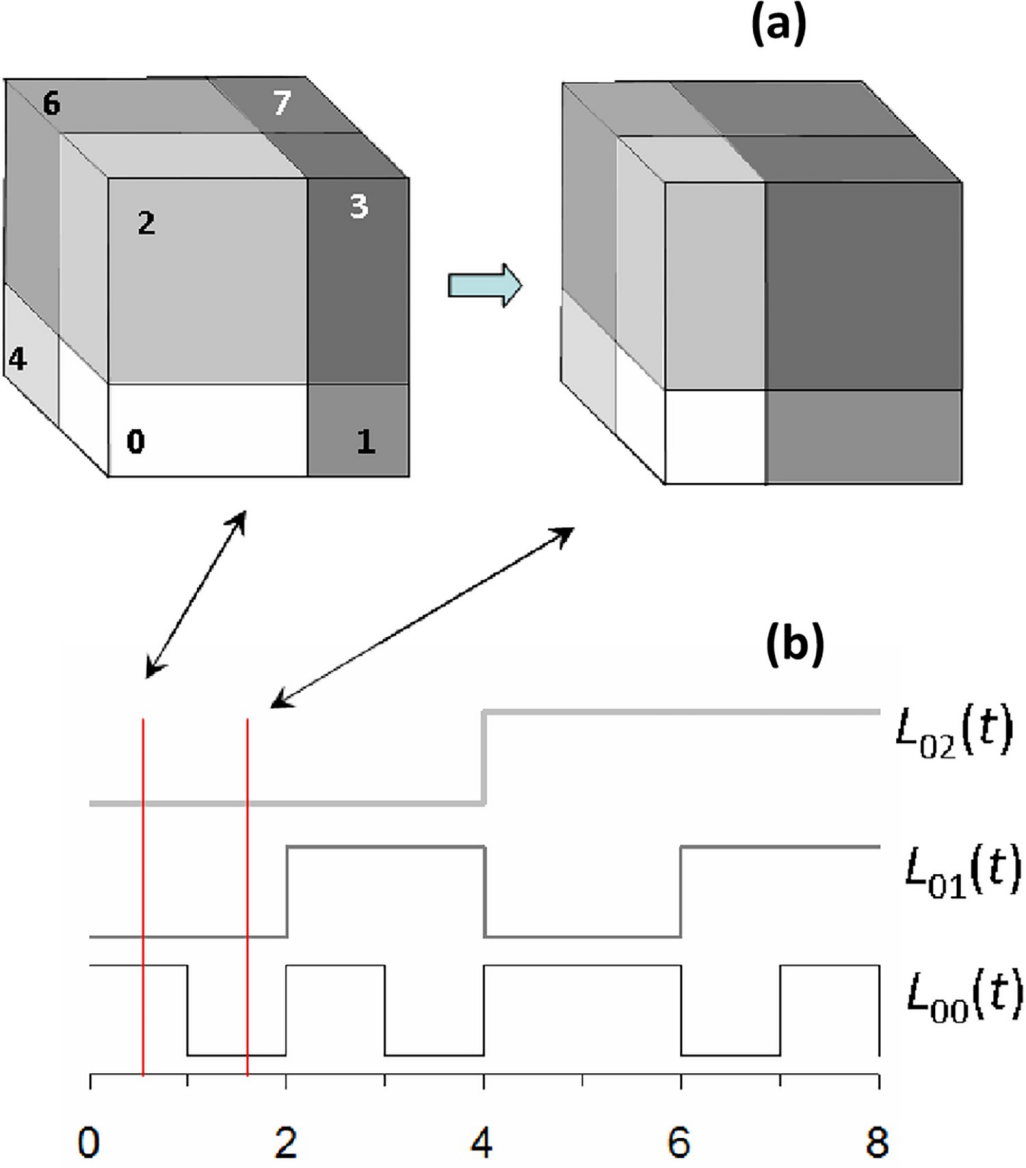
Description of the niche hypercube model used in this paper, for an example with just three resources (e.g. temperature, moisture, pH). Each cell of the hypercube (labelled by species number *s*) is a niche since any organism may be adapted either to low or to high levels of a given resource. This example has 2×2×2 = 8 niches. The volume of a cell determines the carrying capacity for the species in that niche. The total capacity is constant. Each niche volume is determined by the position of the line that divides each axis, which represents the state of the environment. It can have only two states—high or low. Thus, the near lower left box is for an organism that likes all resources low (cell “000”). The dynamics of the environment are modelled by changes in the state of each resource, which changes or holds its state at random, but on its characteristic timescale. In this example, the timescales are one time unit for the horizontal axis, two for the vertical axis and four for the third axis. The upper panel (a) shows the hypercube at two consecutive time-steps. Lower panel (b) shows the changing states of three different resources for cell 0. Here, there is a change in favour of organisms that like high Resource-0 at the expense of those that like low levels of resource-0, while both other resources unchanged.

The community model used in this paper is described in detail elsewhere [40]. This is a niche-based community model which builds on Hutchinson’s *n*-dimensional hypercube model of the ecological niche [41]. In contrast to the model of Hubbell [32], each species occupies a fixed niche with abundance responding entirely to the environmental pressures on that niche. In this conception of the community (Fig 1), we assume a species’ niche size determines its carrying capacity. The product of its share of each resource axis determines this size. For simplicity both resource axes and species are bipolar. For every species that likes a higher value of a given resource, we assume there is another that prefers a lower one. Each axis fluctuates between two values on a different timescale. One axis changes every year, the second every two years, the third every four years, then 8 years, 16 years and so on—thus there is variability on all scales of time and leads to increasing variance with observation time.

There are *K* axes and hence *S* = 2^*K*^ niches for species. The carrying capacity for population of any species is the product of its share of each of the resource axes. The abundance of resource *k* for species *s* is *L*_*sk*_, which is a random outcome of environmental pressure that is only “high” or “low”. Resources change in a stepwise fashion according to their associated timescales. In this study, we will assume that all axes are the same except for the (Special case B in [40]). Thus, we have:

$$\log(L_{sk})=\gamma \pm b$$ (1)

Here *γ* is a measure of the average size of the niche while *b* (|*b*|<1) is a measure of the asymmetry of the environment. For each species, each resource log *L*_*sk*_ has the same long-term variability *b* and the same average value *γ* but different timescales of fluctuation. The resource *L*_*sk*_ is a measure of the width of the niche along the axis *k*. Thus, at any time, the population of a given species *s* is the product of these resources *L*_*sk*_ over the different axes,

$$X_{s}(t)=J\cdot \underset{k}{\prod }L_{sk}(t)$$ (2)

Here *J* is the total community population size (sum of all species populations in the community). If we assume that populations follow changes in the niche size, we can show [40] that the log-population of any species, *s*, is:

$$x_{s}(t)=x_{0}+\overset{K-1}{\underset{k=0}{\sum }}c_{sk}a_{k}(t)$$ (3)

This equation sees the population dynamics as arising entirely from environmental perturbation of the niche for each species. The matrix **C** is a *S*×*K* dimensional matrix with fixed structure (the first column is [-1,+1,-1,+1,-1…], the second column is [-1,-1,+1,+1,-1…] and so on) describing the responses of species to different resource fluctuations (positive or negative). The vector **a**(*t*) (with elements ±*b*) describes the temporal stochastic resource fluctuations on the different resource axes. The constant *x*_0_ = ln*J*+*Kγ* where *J* is the total community size, or carrying capacity. In spite of its complexity, this is a relatively parsimonious community model, since it only uses the parameters *J*, *K*, and *b* (from which *γ* is derived).

### Lognormality in species abundance and time series

Species vary in numbers partially in response to demographic stochasticity but even more as a response to environmental stochastic factors with species that vary most being at greater risk of extinction [23, 44]. This model generates a lognormal species abundance distribution with log*N* having a mean ln*J*+*Kγ* and a variance *Kb*^2^ [40]. Also, the trajectories of abundance for each species through time have exactly the *same* lognormal distribution. The lognormal interpretation has often been criticized for not having a mechanism [45, 46], although several authors have derived theories based on ecological principles leading to a lognormal distribution [38, 47]. Within real communities the distribution of species abundances is often close to lognormal [48].

### Variance growth

This model includes the “more time more variation” effect [49]. The variability of real ecological populations increases over time [50, 51]. This effect means that even species that are very abundant can eventually drift to extinction in the future. A series of studies that systematically examined the relation of variance to time using the Global Population Dynamics Database, supported the idea that variance increases linearly with the logarithm of time as expected for a 1/*f*-noise process [52]. The flexibility of our model allows this pattern to be built into it, so that all populations in the model community fluctuate as 1/*f*-noise. Following our earlier paper [40], we model this 1/*f*-noise by introducing variability in time in all axes of the hypercube, each on a different timescale. We will assume that timescales are equally spaced logarithmically, such as correlation time is proportional to 1, 2, 4, 8, … etc time units. In this framework, the variance of a population time-series of length *T* can be shown [40] to be:

$$V(T)\approx V_{0}\log_{2}T=\frac{b^{2}}{\ln2}\ln T$$ (4)

The result contrasts with the predictions of Hubbell’s neutral model, which predicts a linear increase of variance with time [53] and also with the more traditional population dynamic models (based on logistic model, for example) that assume variance to remain fixed.

### The zero-sum property and extinction

If we add all populations together, we get a constant value of *J*, so all changes add to zero. Likewise for log populations, for which the sum is (ln*J*+*Kγ*)*S*. If any resource changes state, half of all species benefit while the other half suffer. At any time, there is one species well-adapted to everything and there is another badly adapted to everything. These hold if there are no extinctions. Extinction happens if the population of a species falls below some specified minimum viable population (MVP). Once a species becomes extinct its niche remains empty and the zero-sum properties no longer hold.

### Modelling strategy

Our overall strategy is to extract the parameters from the datasets we use and then to forecast by running our community model in repeated simulations. In the case of forest fragments, the model and analysis assume that the fragments are effectively isolated. This assumption is expected to work well for larger patches. In practice, there is some immigration from a larger set of connected patches and nearby contiguous forest areas, which can be expected to make up a significant portion of the birds present on smaller patches.

Unlike some earlier community models that explored extinction dynamics after habitat loss without considering the distribution of species abundances in the community [1, 31], for both cases we estimate the species abundance distribution from our datasets, using these as initial values for the simulations. In both cases, the essential structure of environmental variability is assumed to be 1/*f*-noise whose parameters are found from the GPDD. We will find that the results for the two cases differ radically.

### Parameterization of species abundances: Manaus forest fragments

A set of experimentally-established fragments north of Manaus, Brazil [3, 9, 10] consisted of 11 isolated patches of rainforest, two of 100ha, four of 10ha and five of 1ha. Records consisted of observations of all bird species on these 11 fragments for 14 consecutive years, from 1979 to 1992. This resulted in a total of 14,266 records involving 173 species of bird. Most of the fragments became isolated in 1983; we assume that 1982 represents the initial community.

We fit the model in the following way. We constructed the species abundance distribution for 1982 for the ensemble of fragments and assumed that the total number of species found here (105 species) is *S*_0_, the initial number of species prior to area loss. We also ignored immigration and thus do not include in the analysis species that turn up later. First, we choose the fragment size (1ha, 10ha or 100ha). Following others [54, 55], we assume the number of birds that this kind of tropical forest can support 10 pairs/ha. This means that the fragment population is 20, 200 or 2000 individuals respectively. To get the initial distribution of birds for each simulation, we sample this population from the species abundance distribution. We assume the distribution in Fig A1 in S1 File prevails. We then run the model and observe the evolution of the community, noting the numbers of extinctions as a function of time. The value for the parameter *b* is fitted. We compare this with observed species numbers, for which we counted the species in each fragment as a function of the time since isolation, finding the average number for each size-class of fragment.

### Parameterization of species abundances: British birds

To explain the decline in range or population of birds in the UK [13], there seems to be no single mechanism, though a most commonly cited reason is changing agricultural practices—intensification or abandonment. In Europe, the hardest hit species have been farmland birds. They have fallen by over 50% over 27 years; other groups such as forest birds have suffered only modest declines [56]. Dolton & de Brooke [57] argued that the biomass decline for British birds was in the order of 29% (10% if the semi-domestic pheasant, *Phasianus colchicus*, was included in the calculations) between 1969 and 1988. If we assume all species experienced losses equally, then this is equivalent to a loss of numbers of 15.3% per decade. In this paper we will assume the same declining pattern for all species. A fuller treatment should account for uneven distributions of decline between species [58].

To get the initial distribution of birds, we use the survey quoted by Williamson & Gaston [45] which they regard as a complete enumeration—one of the few studies to do so over large geographical areas. This distribution is consistent with *S*_0_ = 217 and *J* = 130 million. This means that the overall density of birds in Britain at the time of the survey was approximately 3.11 pairs/ha.

### Parameterization of environmental variability

**Fig 2:**
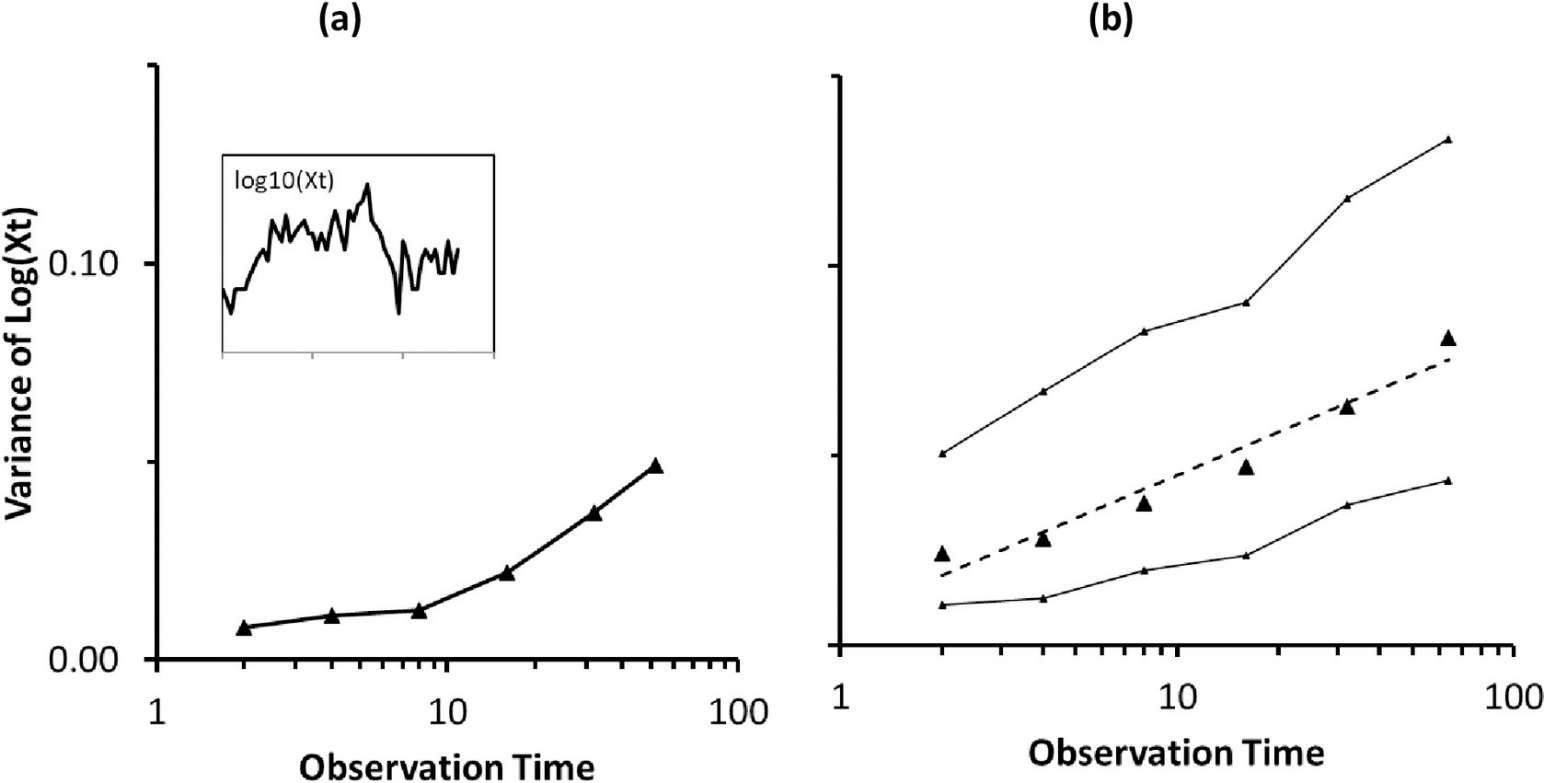
Variance increasing with observation time in the logarithm of ecological populations from the GPDD. This increase tends to proceed as the logarithm of observation time (v~logΔ*t*). (a) An example of variance increase for a bird species (Downy woodpecker, *Dryobates pubescens*) in the GPDD (inset shows dynamics of log-population). (b) Median variance increase found for 63 avian time-series from the GPDD (methods described in [51]. The thinner loci in (b) correspond to the upper and lower 25 percentiles and the broken lines is regression line for Eq (4).

The parameter *B* is fundamental in the model and dominates the probability of extinction in both scenarios that we examine. This parameter should be found from population variability, which in this model reflects environmental variability. In our model, B can be estimated by estimating the variance of ecological time series, for example by using the Global Population Dynamics Database (GPDD) [59]. In earlier papers [52], we used this to investigate variance growth, the so-called “more time more variation” effect. Here, following that approach we estimated the variance growth for all avian time series, limiting our analysis to series with more than 30 points (63 series). We measured the best fitting model of the form *V = V*_0_log_2_(*T*) for the variance growth observed. Then, using Eq (4), we can find *b =* ln*B*, which is the only free parameter. Fig 2 shows in the GPDD [52], variance increases as we observe ecological populations for longer. This agrees with Eq (4) which expects variance (*V*) to scale as logΔ*t* approximately. For birds, for segments of length two years (three points in annual series), the median variance of log*X*_*t*_ is 0.024, that is with a standard deviation of 0.155, which corresponds to fluctuations of *X*_*t*_ between 70% and 143% of the mean. For segments of length 64 years the variance rose to 0.081, corresponding to fluctuations in a range 52% to 192% of the mean. Fitting these data to the Eq (4) above leads to estimates of *B* = 1.11 for birds. These numbers correspond to variance of log*X*_*t*_ increasing by 0.011 for every doubling of series length. It is obvious that as series gets longer the chance of extinction increases. The values of *B* estimated from the GPDD turned out to be too small to explain the levels of extinction of birds observed in the data for the Manaus forest fragments. In order to explain these extinctions, values of *B* = 1.2 (for 1ha fragments) or *B* = 1.5 (for 10ha and 100ha fragments) had to be used. This could either mean that environmental variability for tropical systems is higher or that other mechanisms are playing a role. This was not the case when considering variability under conditions of continuous habitat or population loss for British birds.

### Simulations

In our analyses of the two problems described, once we had chosen a model community, we used the species-abundance distribution, to estimated *S*_0_, the number of species, or *J*_0_, the number of individuals, or both.

For the Manaus forest fragments we chose *K*, the number of axes, so that the species capacity of the hypercube (2^*K*^) would be at least as large as the total number of species observed *S*_0_ (2^*K*^≥*S*_0_). This allows the construction of the basic hypercube for the community prior to habitat losses. To model the initial distribution, we choose random configurations for all axes, which determines the niche hypervolumes for the species. This does not affect the overall community population, which must add up to *J* individuals. The SAD of the species in this community is fitted to the observed species abundance distribution. We assigned species to niches in the hypercube on a hierarchical basis: larger niches are assigned to species with larger observed populations. If there are more niches in the hypercube than *S*_0_, the smallest niches are assigned zero population (unoccupied). For the communities following fragmentation, this is also used to model each fragment following isolation. The event of habitat loss and fragmentation generates a model where all niches to shrink in volume, to that associated with the known fragment area (1ha, 10ha or 100ha). The total number of birds (community population) in each fragment, *J*_0_, is calculated as the area times the known population density of birds in a tropical forest [54, 55]. These are distributed to the niches according to the niche volume. Thus, the initial number of individuals in each niche is proportional to niche-volume, according to Eq (2). When fewer than 0.5 individuals are in a niche the population goes extinct.

For the UK birds, the procedure was much the same. To model the initial distribution, both the initial *S*_0_ and *J*_0_, were based on the species abundance distribution. As time proceeded, the size of the total number of birds in the community became smaller. This decline in carrying capacity is modelled, through Eqs (2–3), by making *J* smaller at each time step, causing increasing numbers of species to fall below the MVP.

In both cases we ran the model to simulate 250 years of time. Extinction could happen in any niche if the population was less than the minimum population size, MVP = 2. There was no re-colonisation. For each parameter set, we ran these simulations 1000 times. We implemented this in the C++ programming language.

## Results

### The loss of species over time from forest fragmentation

**Fig 3:**
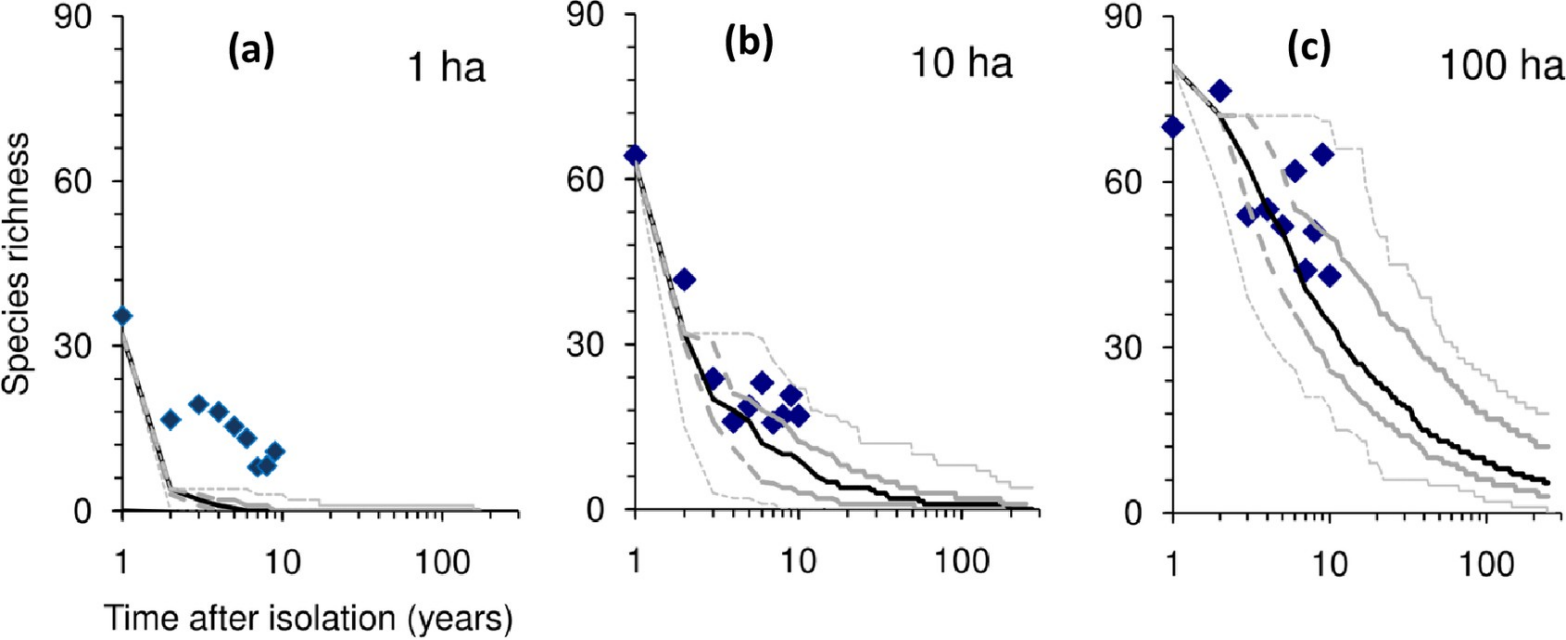
Results for model simulations fitted to the data from Manaus corresponding to three different fragment sizes: (a) 1ha, (b) 10ha and (c) 100ha. The filled diamonds represent observed number of species remaining after a given lag. Each one represents the average number of species seen in a fragment of given size at that lag. The upper and lower dotted lines are minimum and maximum respectively from the 1000 simulations. The heavy dashed lines are the upper and lower 25% (hinges) while the black curve is the median.

Following Preston and others [60–63] we employed a species abundance distribution with octave classes (1,2–3, 4–7, 8–15, etc.) to describe community structure. For the Manaus Forest fragments, this distribution (see Fig A1(a) in S1 File), shows that many species have just 1–3 individuals. This suggests that our data is not a enumeration of the full community, and that some species were just vagrants from other communities. Earlier work [9] analysed these data in some detail. Fig 3 shows the average species richness as a function of time observed for fragments of three different size classes. In each panel the results of simulations are shown for comparison. In all simulations, it was difficult to fit the observed pattern for 1ha. This obviously is related to the fact that such a small fragment can only itself support about 20 individuals and therefore the large numbers of species observed cannot be living entirely within the fragment itself.

On longer timescales, the decline of species richness in this model follows a logarithmic decline. Note that the horizontal axis is logarithmic, so the process of the slowing of relaxation is even greater than is apparent from the visual effect.

To fit the graphs in Fig 3 the parameter *b* had to be large to yield the extinctions observed.

### Loss of species associated with slow declines of carrying capacity

**Fig 4:**
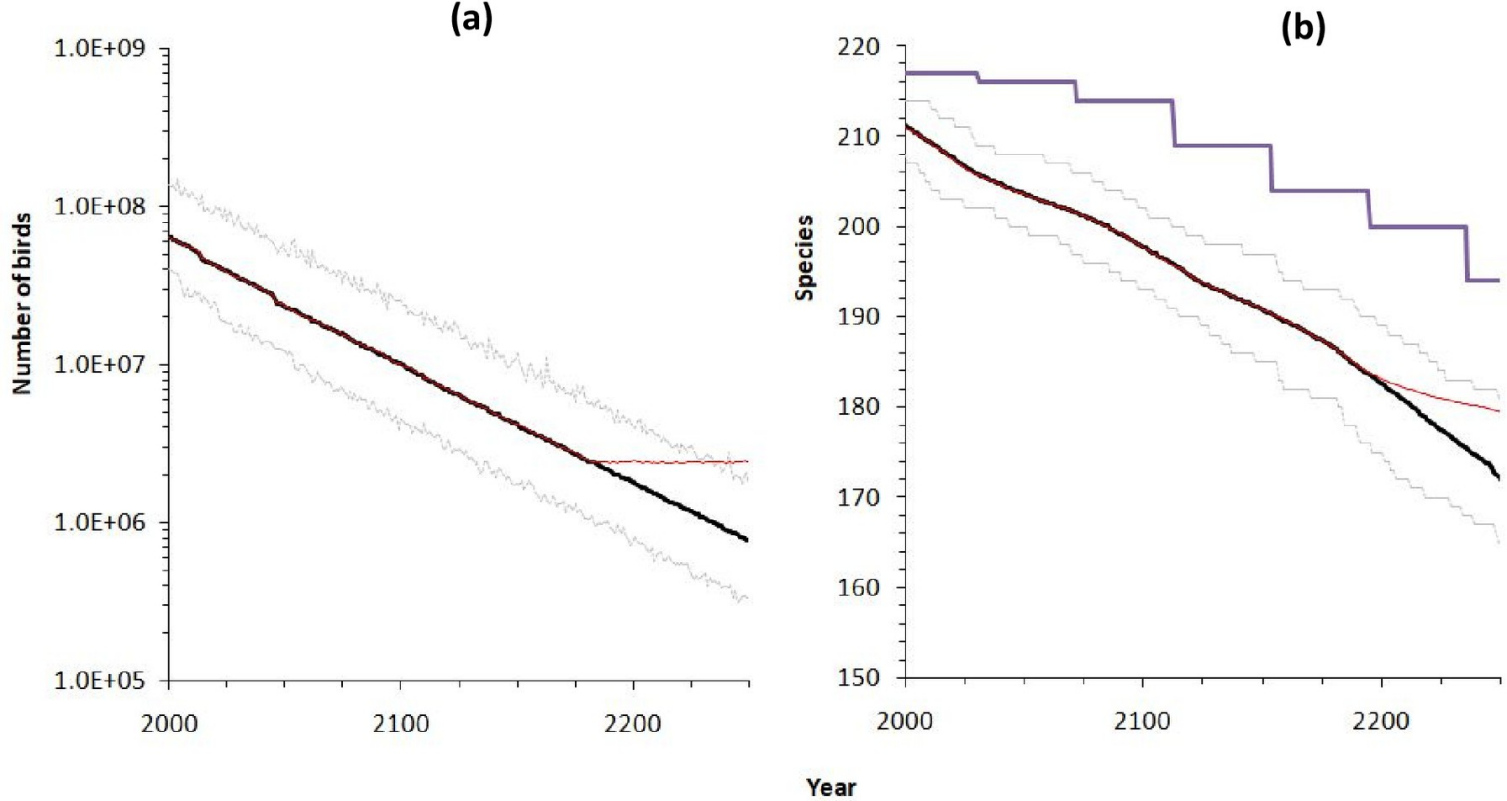
Results for 1000 model simulations fitted to the data for British birds. Left panel (a) shows the quartiles of the total number of birds (over all species). The right panel (b) shows associated species richness. The upper and lower dotted lines are minimum and maximum respectively while the black curve is the median. The staircase pattern in the right panel is the locus of species richness if the species in the lowest octave are lost every time the total population is halved. The red lines show what happens if the loss of population is halted after 200 years, without actual restoration of the population.

Fig 4A shows a continuing decline in the numbers of individual birds if we extend it into the future at the current rate. The initial population is approximately 130 million in 2000, after which the slow deterioration of the environment expresses as an exponential decrease in carrying capacity. As expected, the total number of individuals tracks this decline, falling to less than 1.88 million after 250 years. This decline, if it continued would lead to the eventual disappearance of all birds within 1100 years.

Fig 4B shows the corresponding species richness. Note that the vertical axis is not logarithmic so the decline of species richness is almost linear. For the first 50 years or so biodiversity declines at 1.3 species per decade. Thus, half the species would be gone by the year 2500. In contrast to the previous scenario with forest fragments, here the rate of extinction begins at a small value and then reaches a maximum when approximately half the species have been lost. A direct calculation using the species-abundance distribution alone (ignoring dynamic effects, see Appendix in S1 File) yields an estimate of 0.37 species per decade, underestimating the initial loss of species.

Both panels also show what happens if the loss of population is halted after 200 years, without population being actually restored. This is clearly visible in Fig 4A, while in Fig 4B we see that the decline in species richness slows but does not stop. Thus, there is still a major extinction debt in play here.

## Discussion

Our objectives are to use a dynamic model to project species losses into the future in two scenarios. In the first scenario, we consider observed species losses in forest fragments over a short period and then forecast longer-term consequences. In the second, we consider the long-term consequences of a steady proportional (i.e. exponential) loss of individuals from a community.

### Forest fragments

**Fig 5:**
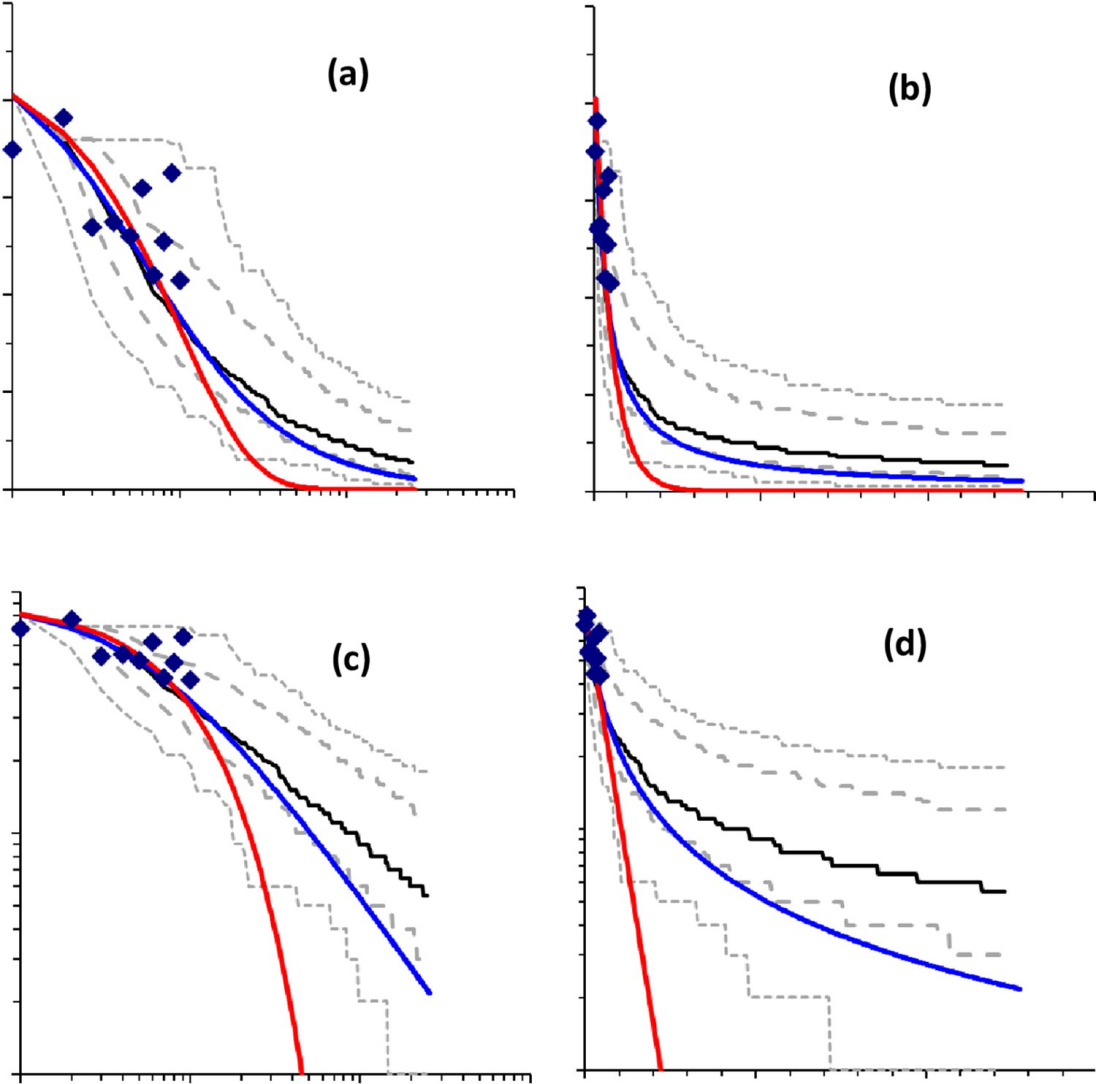
Survival curves for the 1/*f*-community model corresponding to Fig 3C (100 ha fragment) repeated on a larger scale and using all four different combinations of log-linear axes. Displayed are the median, upper and lower 25 percentiles and extremes for 1000 simulations as well as the observed data. The left panels (a,c) have logarithmic axis for time, while those on the right (b,d) use a linear time axis. Upper panels (a,b) have a linear axis for species richness, lower panels (c,d) have a log axis. Drawn also are an exponential model of decay (red line) and a hyperbolic model (blue line), both with the same half-life *t*_50_.

Fig 5 emphasises that relaxation is a multi-scale phenomenon in time. It shows also the behaviour of biodiversity loss under different models. The community 1/*f* model is compared to an exponential decline or a hyperbolic decline expected for a neutral community [31]. In all cases, we have also fit two curves showing exponential declines (red) and hyperbolic declines (blue, corresponds to neutral model). These curves are equivalent in that they have the same half-life *t*_50_. To better show the model’s features, we use the four combinations of log and linear axes for both time and the number of species.

At the scale of our observations, there is little difference between the various models. The issue is what happens at longer scales. And for this, at least for the data shown, a linear time axis is not appropriate. Conversely, a linear diversity axis shows more clearly what is going on than a log one. This is what we expected as the dominant dynamics of 1/*f* noise is that variance increases with log of time. Simply, the log-time, linear-diversity plot–top left–is more informative.

Compared to both fitted declines, but especially, the exponential decline, the declines predicted by the model involve very much slower losses of species in the long-term. In short, the relaxation is rapid at first but slows down, so that even after 100 years, a fragment of size 100ha continues to lose species. The delay tends to increase with area. This pattern agrees with earlier models using pure neutral theory [31], some modification of neutral theory or alternative schemes [1, 26] that also yield increasing delays with area. This analysis also reveals that isolated fragments suffering relaxation cannot be the whole story. For very small fragments, smaller than expected species decline suggest that vagrants or fugitives from elsewhere played an important role. This can be seen in Fig 4A, where species richness remains higher than 10 on 1ha fragments, even though the numbers than can be supported by such small fragments are about 20 birds. Conversely on large fragments, smaller than expected species richness suggests that environmental conditions were changing more than expected or that populations were in a more neutral phase. It is also possible that at the same time, the proximity of larger forest areas can act as a “magnet” drawing birds away from fragments.

### Continuous habitat loss

The case of continuous habitat or population loss presents a very different picture. Our investigation on the steady loss of the population of British birds assumes a loss of 15.3% of individuals per decade. Our analysis of this topical issue leads to a prediction of a rather steady loss of 1.3 species per decade for the near future (until 2300) followed by a more rapid decline. Thus, at this rate, several centuries would pass before a significant proportion of species were lost and so we expect this rate of species loss to be insignificant compared to stochastic factors and conservation intervention. Indeed, since 2000 in the UK, fewer species have been lost than have been reintroduced. In contrast to the case of isolated fragments, extinction debt plays a less visible role. This is because the dynamics of extinction debt are confounded by the dynamics of declining population. If we include a brake on the process of exponential decline at some fraction of current population, we see a continued but slower species decline. Thus, the dynamics are always present and sometimes complex. A simple approximate way to view this is through the octaves of the SAD (species abundance diagram in Appendix: Fig A1 in S1 File). Each time the number of individuals in the community is halved, the diagram must move one octave to the left, with the leftmost class going to extinction. This approach yields a similar pattern but it underestimates the initial number of extinctions.

Thus, in cases of slow declines of habitat or population, such as for the declining British birds, we do not predict extinctions to happen quickly and there is plenty of time for conservation action. However, this is not expected to be always the case. For example, a more alarming report is in the case of invertebrates, where a loss of 75% of biomass occurred in protected areas in Germany over the last 27 years [16], equivalent to a half-life of 13.5 years. Losses on this scale are by contrast likely to lead to more significant biodiversity consequences on foreseeable timescales. By using a plausible SAD based on Preston’s canonical lognormal hypothesis others [60–63] we can infer a species abundance distribution for the corresponding insect community. Using this hypothesis (Appendix in S1 File) we estimate the number of individuals needed to support a diversity of 20,000 species is approximately 5×10^16^ (Appendix in S1 File). The analysis of this community with these assumptions and given rate of population declines, leads to species losses that are relatively flat for another few decades (the numbers of individuals are so large) but it increases rapidly after 50 years. Thus, even with the large losses of insects observed, the initial extinction rates seem small. But this is deceptive since they increase exponentially thereafter and even if the loss rate of halted, there will be a large extinction debt.

### Conclusions

(1) The analysis of biodiversity loss from different types of ecosystem suggests that the dynamical aspects are always an important consideration. Our analysis here, using a niche-orientated community model reveals similar patterns of relaxation and extinction debt as found with neutral models. So, extinction debt is not an artefact of neutrality models examined previously.

(2) From habitat fragments, we typically see the rapid loss of many species followed by a slower loss of species on longer timescales. This provides an explanation of the possible paradox of relaxation times in years in experimental habitat fragments and many millennia for islands created by Pleistocene sea-level rise. It warns that the rapid loss of species might not continue in the medium term, but equally that because species losses have slowed, they will nonetheless continue.

(3). More speculatively, we look at what is going to happen given the large current losses of population sizes in continental areas, we find that initial extinction rates are not expected to be very large and may go undetected. However, rates of decline on larger timescales will be massive and subject to extinction debt. Much the same may be true for populations in lowlands and northern species at the southern edges of their ranges. Their current survival does not mean continued survival in the long-term.

## Supporting information

S1 File Octave-based method of calculating extinctions. (DOCX)

S2 File Fig 2 and data used to draw it. (XLSX)

S3 File Fig 3 with data and calculations used to draw this figure. (XLSM)

S4 File Instructions on how to use S3 File. (DOCX)

S5 File Numbers used to draw Fig 4 (for UK birds data). (XLSX)

S6 File Program used to generate numbers in S5 File. (EXE)

S7 File Input instruction file for the program WinC1f.exe (for UK birds data). (DAT)
