# Supplementary material for: The rate of species extinction in declining or fragmented ecological communities
Source: PLoS One. 2023 Jul 12;18(7):e0285945. doi: 10.1371/journal.pone.0285945 (PMC10337920; doi:10.1371/journal.pone.0285945)
Supplement: S1 File — (DOCX) [file pone.0285945.s001.docx]

**TITLE: The rate of species extinction in declining or fragmented ecological communities**

**AUTHORS:** John M. Halley and Stuart L. Pimm

**SUPPLEMENTARY INFORMATION**

**Appendix.** **Octave-based method of calculating extinctions**

Suppose biodiversity declines at a rate of *x* species per decade. Thus, half the species would be gone by the year 2500. A direct calculation using the species-abundance distribution alone and ignoring dynamic effects within the community is possible by observing the species abundance distribution (Fig A1).


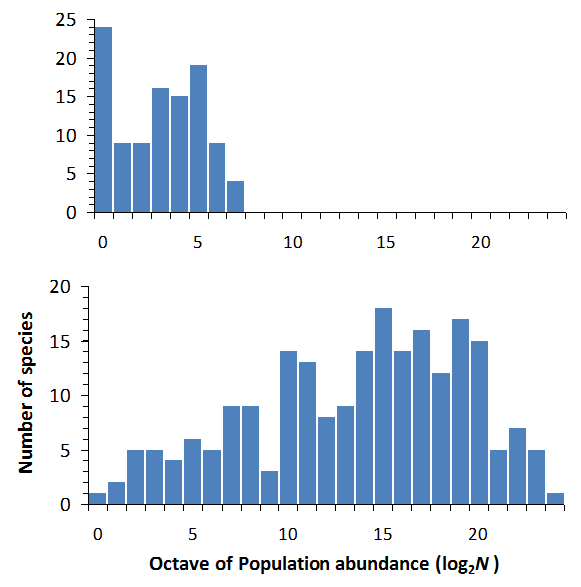


**(*a*)**

**(*b*)**

***(a)***

***(b)***

**Figure A1**. (a) Species abundance distribution of birds for the Manaus system caught in the early stages of the experiment. We assume that these species represent a representative sample of the species in the area prior to the isolation of fragments. This distribution contains 105 different species and 2142 individuals. Following Preston, we use a log2 scale, or octaves. (b) The corresponding SAD for British birds based on data quoted by Williamson & Gaston [1].

**Method when we know the SAD**

The method proceeds in the following way. We assume that the Species Abundance Distribution (SAD) has values *y*_0_, *y*_1_, *y*_2_, *y*_3_, … *y_k_* at octaves 0, 1, 2, 3, …*k* with centres at 1, 2, 4, 8, …2*^k^* respectively. Thus, the total population is

$J\approx\sum_{j=0}^{k} y_{j}2^{j}$ ...(A1)

Suppose overall population is declining according to an exponential equation

$J(t)=J_{0}e^{-t/\tau}$ ...(A2)

This means that every $t_{50}=\tau\ln2$ years the overall community population *J*(*t*) falls to half its previous value *J*(*t-t*_50_). If the decline is shared by all species equally each class in the Preston diagram above moves one octave to the left. The species in octave-0, all *y*_0_ of them, go extinct. In another *t*_50_ years, the species now in octave-0 go extinct, and so on. Assuming no redistribution of relative population sizes, the total species number follows a declining trajectory given by:

$S(t)\approx S_{0}-\sum_{i=0}^{\mathrm{int}\left(t/t_{50}\right)} y_{i}$ ...(A3)

**Method when we don’t know the SAD**

In the case where we do not know the SAD, we can use the Canonical Lognormal hypothesis of Preston [2] following other researchers [3]. It is well-known that the Canonical Lognormal Distribution (CLD) is not always a good approximation. However, it is one way of constructing a plausible distribution beginning with little information, either the number of individuals or the number of species. In the event that we know the number of species, the method proceeds as follows.

Suppose the SAD is given by the normal distribution where the octave number is given by R:

$y(R)=\frac{S}{\sigma\sqrt{2\pi}}e^{-R^{2}/2\sigma^{2}}$ ...(A4)

Thus, we do not work with the entire range of possible octaves R∈(-∞,∞) but with the reduced range *R*∈[*R*_min_,*R*_max_] defined by the finite community size. The number of nonzero octaves is determined by the ratio of the commonest to the rarest species. The total number of octaves *R*_max_ is determined by criterion that we miss at most half a species on either side:

 ...(A5)

Where we have assumed the distribution is symmetric *R*_min_=-*R*_max_, and used the symbol *φ*(*x*) for the cumulative standard normal distribution:

So that

 ... (A6)

Preston claimed that for many real communities the distance between the peaks of the SAD and the Individuals Curve (*Y=*2*^R^y*) was simply *R*_max_, that is the individuals curve reaches its peak when *R=R*_max_, which means that:

$R_{\max}=\text{ln2}\cdot\sigma^{2}$ ... (A7)

Thus, combining (A6) and (A7) we have:

 ... (A8)

Thus the st. dev. *σ* can be calculated from species number *S*, and *R*_max_ can be calculated from (A7). This allows us then to construct the entire SAD, which can then be used to give the results in (A1-A3).

**Using Canonical Lognormal Hypothesis to forecast invertebrate losses in the UK**

The findings of Hallman et al of a loss of 75% of invertebrate biomass over 27 years in protected areas, equivalent to a half-life of 13.5 years, will lead to significant invertebrate biodiversity losses on foreseeable timescales. The number of invertebrates is much larger, with 20,000 species in the UK alone, which is too large to run our community-1/*f*-noise model as currently configured. However, our analysis of birds suggests that using a plausible SAD might provide some sense of near-term extinction rates. Preston argued that a specific lognormal predominates in ecological communities — one in which most of the individuals in a community belong to a few of the most abundant species, rather than, for example, to many of less abundant ones. Thus, although we do not have a species abundance distribution for the corresponding insect community, following Preston and others a first guess employs the Canonical Lognormal hypothesis [2,3]. Using this hypothesis, the number of individuals needed to support a diversity of 20,000 species is approximately 5×10^16^ (Appendix). The SAD will thus have approximately 54 octaves. The analysis of this community with these assumptions and given rate of population declines, leads to an extinction curve below in Fig6.

This suggests that species losses are relatively flat for another few decades (the numbers of individuals are so large) but it increases rapidly after 50 years. Thus, even with the large losses of insects observed, the initial extinction rates seem small. But this is deceptive since they increase exponentially thereafter and even if the loss rate of halted, there will be a large extinction debt.


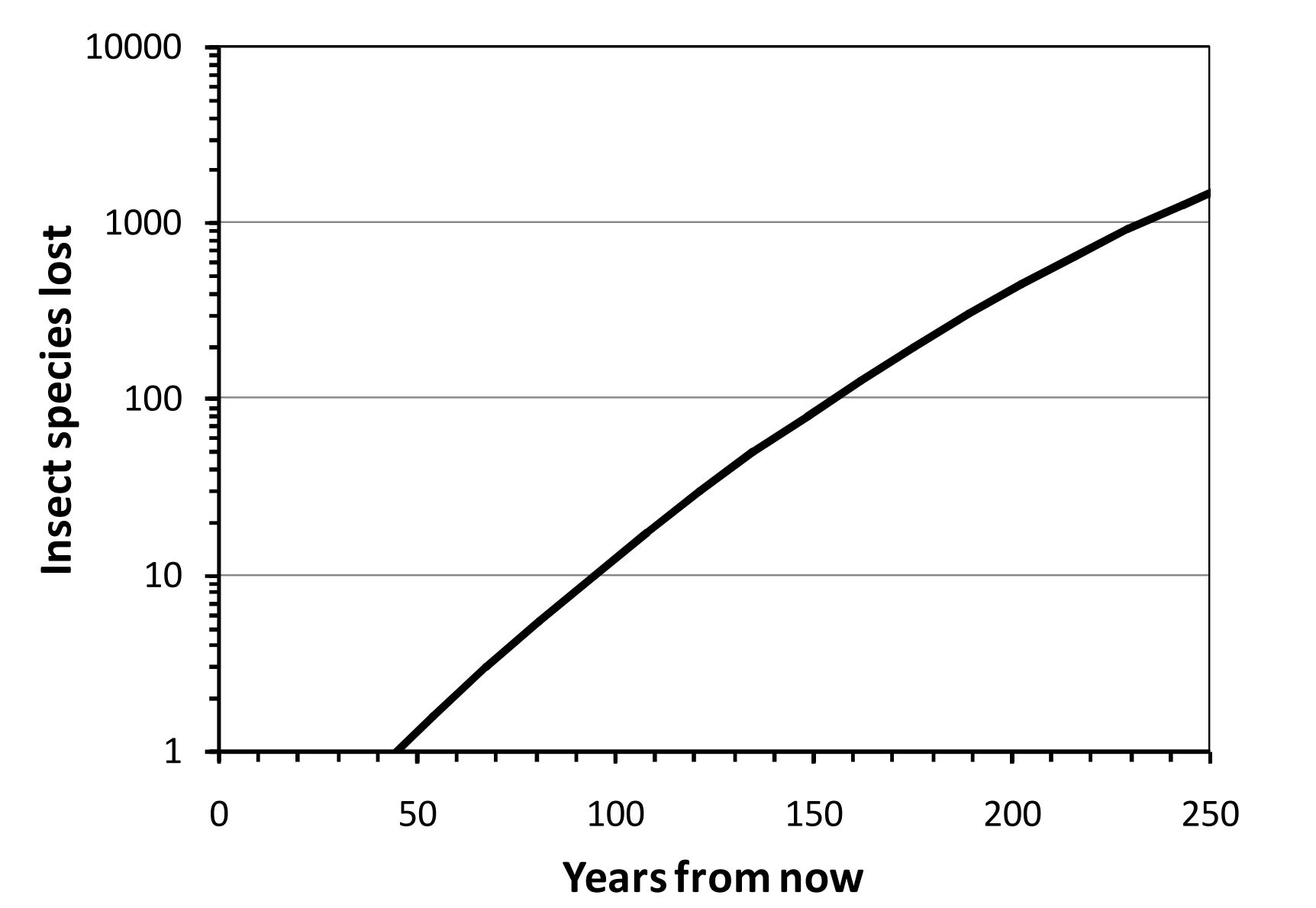


**Figure A2**. Expected initial pattern of extinction of UK insects assuming loss rates similar to those of Hallman et al (2017) and assuming the canonical lognormal hypothesis. Assuming the same rates of loss of individuals or habitat the curve reaches an asymptote of 20,000 after 500 years.

**REFERENCES**

1. Williamson M, Gaston KJ. The lognormal distribution is not an appropriate null hypothesis for the species-abundance distribution. J Anim Ecol. 2005 May;74(3):409–22.

2. Preston FW. The canonical distribution of commonness and rarity: Part 1. Ecology. 1962;43(2).

3. Curtis TP, Sloan WT, Scannell JW. Estimating prokaryotic diversity and its limits. Proc Natl Acad Sci. 2002 May 25;99(16):10494–9.
