# Supplementary material for: The rate of species extinction in declining or fragmented ecological communities
Source: PLoS One. 2023 Jul 12;18(7):e0285945. doi: 10.1371/journal.pone.0285945 (PMC10337920; doi:10.1371/journal.pone.0285945)
Supplement: S4 File — (DOCX) [file pone.0285945.s004.docx]

**EXPLAINING SIMULATIONS FOR FIG3**

**Niche vol. sheet**

This sheet creates niche volumes for 128 species for 512 timesteps. It uses the EXCEL random number generator “rand()” for everything. Species are in columns and time is in rows. Columns are used to represent the processes a0-a10 in columns B-L but in orientation matrix the processes are associated with rows 10-28 while columns O-BZ represent the 128 species.

An Initial Value Spectrum is specified initially for the different processes. This was set to [1,1,1…,1] without loss of generality. This is set in C5-I5.

There are 10 processes with 10 axes called a1-a10. This allows for up to cycle times of 1,2,4,…,512 (row 8). Each of these has a value ±1. Process a1 is always a random variable with no memory using “=2*INT(RAND()+0.5)-1”, while the others can change state (or not) after durations equal to their cycle times. The states of these RVs are stored in cells C10-C521, while times 1-512 are listed in column A10-A521. The random variable a0 represents processes that happen on timescales shorter than one time unit, the magnitude of which is controlled by the parameter blues. It is also generated by =2*INT(RAND()+0.5)-1 and is multiplied by blues defined in control sheet. Cells that change on pressing F9 are colour-coded orange. White cells are linked to these and follow them.

We model 128 species individually, representing a total of 7 axes. The orientation matrix C has dimensions 7X128 and is found in two blocks: in O10-BZ17 and O22-BZ28.

The matrix multiplication yields a row of 1X128 for each time-step, since time is in rows. Then there are a total of 512 rows, thus populating cells CF10-HC521.

Each cell contains the formula

$$=blues*a_{0}(t) + \sum_{j=1}^{7} C_{sj}*a_{j}(t) + reds*\sum_{j=8}^{10} a_{j}(t) + lonewite*N_{st}(0,1)$$

The term with reds refers to the external imposed LF noise that changes independently and affects all species equally and reds is the strength of this. The last term is an imposed HF noise that changes independently of species and affects each species differently. The parameter lonewite is the strength of this effect.

**Control sheet**

This sheet contains most of the control parameters and also significant outputs.

In the top right corner (cells A1-J30) are defined the order of the community, typically K=7. Also, is the very important parameter of asymmetry, B, and all the parameters derived from this: b, γ and Γ. On this sheet is also the size of MVP and the community population ceiling/capacity, J. On this corner also are the decisions on the habitat destruction: when does it happen, when is habitat restored etc.

At any one time, the file produces an output of one simulation of time 0<t<512. These results, species richness as a function of time, are shown in columns K,L. Column M shows observed diversity and column N shows the deviations and RMSD for the current simulation.

By the use of a macro we can generate repeats of the worksheet calculations and hence generate many replicates. This is done in Row 20, starting in column P. In this row we can put both inputs and outputs, so the macro puts the results in rows 21 and following, so as to build up a set of replicates for given parameters.

On 20/2/23, we carried out 5000 replicates, choosing both B and blues at random from intervals [1,2] and [0,10] respectively. For J=200 (10ha), we found that the lowest RMSD was when 1.4<B<1.6, with blues=0 and low values we also obtained when 1.2<B<1.3 with blues=1. For J=2000 (100ha), we found that the lowest RMSD was when B=1.9, with blues=0. For B=1.2, the best RMSD was with 1.0 for blues.
